# Dysfunction of the Hippocampal-Lateral Septal Circuit Impairs Risk Assessment in Epileptic Mice

**DOI:** 10.3389/fnmol.2022.828891

**Published:** 2022-04-29

**Authors:** Yi Cao, Chongyang Sun, Jianyu Huang, Peng Sun, Lulu Wang, Shuyu He, Jianxiang Liao, Zhonghua Lu, Yi Lu, Cheng Zhong

**Affiliations:** ^1^Guangdong Provincial Key Laboratory of Brain Connectome and Behavior, CAS Key Laboratory of Brain Connectome and Manipulation, The Brain Cognition and Brain Disease Institute, Shenzhen Institute of Advanced Technology, Chinese Academy of Sciences, Shenzhen-Hong Kong Institute of Brain Science-Shenzhen Fundamental Research Institutions, Shenzhen, China; ^2^Division of Life Sciences and Medicine, School of Life Sciences, University of Science and Technology of China, Hefei, China; ^3^Key Laboratory of Industrial Microbiology, College of Biotechnology, Tianjin University of Science and Technology, Tianjin, China; ^4^College of Electronic and Information Engineering, Hebei University, Baoding, China; ^5^Shenzhen Children’s Hospital, China Medical University, Shenzhen, China; ^6^Epilepsy Center, Shenzhen Children’s Hospital, Shenzhen, China

**Keywords:** temporal lobe epilepsy, risk assessment, forthcoming approach, hippocampus, E/I imbalance, lateral septum

## Abstract

Temporal lobe epilepsy, a chronic disease of the brain characterized by degeneration of the hippocampus, has impaired risk assessment. Risk assessment is vital for survival in complex environments with potential threats. However, the underlying mechanisms remain largely unknown. The intricate balance of gene regulation and expression across different brain regions is related to the structure and function of specific neuron subtypes. In particular, excitation/inhibition imbalance caused by hyperexcitability of glutamatergic neurons and/or dysfunction of GABAergic neurons, have been implicated in epilepsy. First, we estimated the risk assessment (RA) by evaluating the behavior of mice in the center of the elevated plus maze, and found that the kainic acid-induced temporal lobe epilepsy mice were specifically impaired their RA. This experiment evaluated approach-RA, with a forthcoming approach to the open arm, and avoid-RA, with forthcoming avoidance of the open arm. Next, results from free-moving electrophysiological recordings showed that in the hippocampus, ∼7% of putative glutamatergic neurons and ∼15% of putative GABAergic neurons were preferentially responsive to either approach-risk assessment or avoid-risk assessment, respectively. In addition, ∼12% and ∼8% of dorsal lateral septum GABAergic neurons were preferentially responsive to approach-risk assessment and avoid-risk assessment, respectively. Notably, during the impaired approach-risk assessment, the favorably activated dorsal dentate gyrus and CA3 glutamatergic neurons increased (∼9%) and dorsal dentate gyrus and CA3 GABAergic neurons decreased (∼7%) in the temporal lobe epilepsy mice. Then, we used RNA sequencing and immunohistochemical staining to investigate which subtype of GABAergic neuron loss may contribute to excitation/inhibition imbalance. The results show that temporal lobe epilepsy mice exhibit significant neuronal loss and reorganization of neural networks. In particular, the dorsal dentate gyrus and CA3 somatostatin-positive neurons and dorsal lateral septum cholecystokinin-positive neurons are selectively vulnerable to damage after temporal lobe epilepsy. Optogenetic activation of the hippocampal glutamatergic neurons or chemogenetic inhibition of the hippocampal somatostatin neurons directly disrupts RA, suggesting that an excitation/inhibition imbalance in the dHPC dorsal lateral septum circuit results in the impairment of RA behavior. Taken together, this study provides insight into epilepsy and its comorbidity at different levels, including molecular, cell, neural circuit, and behavior, which are expected to decrease injury and premature mortality in patients with epilepsy.

## Introduction

Epilepsy is a common brain disorder with repeated seizures and is often comorbid with other neuropsychiatric disorders (Moshe et al., 2015; Kanner, 2016; Hermann et al., 2021). Previous research has shown that epileptic patients have a higher risk of premature mortality due to external causes, including falls and drowning (Fazel et al., 2013). Risk assessment (RA) plays a key role in survival, and its impairment may lead to injury or even death (Gross and Canteras, 2012; McNaughton and Corr, 2018). When there is ambiguity and conflict between the internal approach and avoidance tendencies, RA is likely to arise primarily. Previous studies have suggested that individuals with epilepsy have RA impairment, but the results are controversial (Kubova et al., 2004; O’Loughlin et al., 2014). However, there is little knowledge of the underlying neural mechanisms involved in RA dysfunction in epileptic individuals.

Temporal lobe epilepsy (TLE) is one of the most common and refractory epilepsies in adults and represents pathological alterations in the hippocampal structures (Tellez-Zenteno et al., 2005; Curia et al., 2008; Ren and Curia, 2021). The hippocampus (HPC) performs a broad range of brain functions, especially contextual cognitive behaviors. To obtain information about a novel environment with potential threats and to support the following choice between approach and avoidance, RA is necessary (Gross and Canteras, 2012; McNaughton and Corr, 2018). In a conflict context, for example, rodents in the elevated plus maze (EPM), RA behaviors were observed as crouch-sniff and stretch-attend in the center zone. RA behavior was considered as both a cognitive and emotional process. It is generally considered that the ventral hippocampal neurons participate in the modulation of anxiety behaviors, while the dorsal hippocampal neurons play an important role in coding spatial information (Fanselow and Dong, 2010). Recent studies have revealed that the dorsal hippocampus also regulates mild anxiogenic contextual exploration (Dong et al., 2021) and ambiguous threat context (Besnard et al., 2020). As the HPC and its downstream region represent diverging patterns of coding of RA information, which correlated with subsequent behaviors, including approach and avoidance of the open arm (Jacinto et al., 2016), it should be confirmed whether the abnormal hippocampal network contributes to the impairment of RA in TLE.

In TLE, the HPC of both human patients and animal models showed obvious neuronal loss and network reorganization. Importantly, this alteration shows high neuronal heterogeneity (Lothman et al., 1995; Mangan et al., 1995) which often induces an excitation and inhibition (E/I) imbalance. The E/I imbalance in TLE not only contributes to seizures, but is also related to other brain dysfunctions, such as unconsciousness (Motelow et al., 2015). During seizures, the E/I imbalance, typically as the hyperexcitability of glutamatergic neurons and/or the reduced inhibition of GABAergic neurons, causes epileptic waveforms and propagates from the hippocampus downstream (Krook-Magnuson et al., 2013; Levesque et al., 2016; Lu et al., 2016). Furthermore, selective loss of dentate hilar somatostatin-positive (SOM+) neurons was observed, whereas loss of cholecystokinin-positive (CCK+) or parvalbumin-positive (PV+) neurons was not significant in TLE (Sun et al., 2007; Benini et al., 2011). Therefore, we hypothesized that profound loss and dysfunction of hippocampal GABAergic neurons in TLE may result in an imbalance of E/I and contribute to impaired RA behavior.

Both hippocampal dentate gyrus /hilus (DGH) GABAergic neurons and glutamatergic neurons have been found to play different roles in EPM (Botterill et al., 2021; Wang et al., 2021). Ventral hippocampal vasoactive intestinal polypeptide positive (VIP+) GABAergic neurons can distinguish the open and closed arms in EPM more strongly than PV+ or SOM+ interneurons (Lee et al., 2019). However, the subtype of GABAergic neurons that preferentially respond to the central zone remains unknown. Many hippocampal downstream regions have been suggested to be involved in RA, including the prefrontal cortex, amygdala, and lateral septum (LS) (Xia and Kheirbek, 2020). The LS, in which more than 95% of neurons are GABAergic interneurons, is a vital relay between the hippocampus and other regions and has been implicated in a wide variety of functions (Wirtshafter and Wilson, 2021), including emotional, motivational, and spatial behavior, as well as RA behavior (Motta et al., 2017; McNaughton and Corr, 2018). Dysfunction of the hippocampal-lateral section (HPC-LS) is suggested to be involved in epilepsy (Motelow et al., 2015). Thus, an investigation into the cellular mechanism of E/I imbalance in HPC degeneration in impaired RA is necessary.

Herein, we show that kainic acid (KA)-induced epilepsy preferentially has an impairment of RA. Through extracellular electrophysiological recordings, we found that hippocampal dorsal dentate gyrus and CA3 (dDG/CA3) neurons participate in response to RA, in which a subpopulation of them is specifically activated by approach-RA and another subpopulation is specifically activated by avoid-RA. Epileptic mice show E/I imbalance of dDG/CA3 in the approach-RA, where the approach-RA specifically activated glutamatergic neurons increased, whereas the approach-RA-specifically activated GABAergic neurons decreased. In addition, in the LS, the approach-RA specifically activated GABAergic neurons decreased. Moreover, our results imply that the decreased dDG/CA3 GABAergic neurons are SOM+ neurons, while the decreased LS GABAergic neurons are CCK+ neurons. Taken together, the epilepsy-induced impairment of RA due to dysfunction of the dDG/CA3-LS circuit, which shortens the approach-RA duration. Meanwhile, when they approached the threat with inadequate RA, they were more likely to be injured.

## Materials and Methods

### Animals

Adult male C57/BL6J mice (18–22 g; 6–8 weeks old) were purchased from the Guangdong Medical Laboratory Animal Center (Guangdong Province, China). Adult Sst-IRES-Cre knock-in mice (Jackson Laboratory, repository number 013044), expressing Cre recombinase in somatostatin-expressing neurons, and CCK-IRES-Cre knock-in mice (Jackson Laboratory, repository number 012706) expressing Cre recombinase in cholecystokinin positive neurons, were bred, identified, and provided by the Shenzhen Institute of Advanced Technology using specific criteria (18–22 g; 6–8 weeks old). Animals were housed under the following laboratory conditions: ambient temperature, 24 ± 1°C; humidity, 50–60%; 12-h light/dark cycle beginning at 8 a.m.; food and water *ad libitum*. All experiments were performed in accordance with protocols approved by the Ethics Committee for Animal Research, Shenzhen Institute of Advanced Technology, Chinese Academy of Sciences (SIAT-IACUC-20210617-NS-NTPZX-ZC-A0893-03).

### Fabrication of Electrode Arrays

Microwire electrode arrays, each containing 10 stereotrodes (20 channels), were fabricated from 17.78 μm diameter formvar-coated nickel-chromium wires (California Fine Wire Company, Grover Beach, CA, United States). Each stereotrode was threaded through a silica tube (TSP100170, Polymicro Technologies, Phoenix, AZ, United States). Each stereotrode was wrapped around two adjacent pins of a standard electrode connector (A79026, Omnetics connector, Minneapolis, MN, United States). Silver microwires (*OD* = 200 μm, 99.95% pure) were then soldered to four pins on the outer side of the connector as ground and reference, respectively. Acrylic resin was used for the encapsulation. Electrode tips were plated with platinum to reduce impedance to 300–800 kΩ (at 1 kHz in PBS) before use. The electrode arrays were fabricated as previously described by Sun et al. (2022).

### Surgery

Mice (8-week-old, male) were treated with atropine (0.2 mg/kg) 15 min before the experiment to overcome breathing problems. General anesthesia was achieved by intraperitoneal administration of urethane (1.5 g/kg). After the animal’s head was fixed in a standard stereotaxic frame, the cranium was exposed through a small midline scalp incision. A small craniotomy was performed with a high-speed dental drill, and the tips of the 34-gage needle (Hamilton) were directed toward the brain at the following stereotaxic coordinates: at anteroposterior (AP) −1.8 mm, mediolateral (ML) −2.0 mm, and dorsoventral −1.8 (DV) mm and then 650 nL (0.3 mg/mL in 0.1M) of KA solution was injected into the dorsal hippocampus using a micropump. After 8–12 weeks of injection, the KA-induced chronic temporal lobe epilepsy model was established as previously described (Krook-Magnuson et al., 2013; Moura et al., 2020).

For chemogenetic testing, the virus was carefully drawn up into a 10 μL Hamilton syringe with 34-gage needle by automatically raising the plunger. The virus [200 nL rAAV-EF1α-DIO-hM4D(Gi)-mCherry] was injected into the dCA3 area of the brain (AP, −2.00 mm; ML, ±1.50 mm; DV, −2.00 mm) of SOM-cre mice at a rate of 100 nL min^–1^, followed by a 9 min pause. For optogenetic testing, the virus (300 nL AAV-CaMKII-ChR2-mCherry) was injected into C57/BL6J mice. After 3 weeks of viral proliferation, an optical fiber (diameter, 200 μm) was inserted into the dCA3 area of the brain for optical stimulation. After the procedure, all animals were allowed to recover for a week in their home cages with free access to food and water.

For electrode implantation, a neural electrode was directed toward the brain at the following stereotaxic coordinates after the craniotomy: the tips of the stereotrodes at AP +0.74 mm, ML −0.75 mm, and DV −2.80 mm in a 10° angle for dLS recording and at AP −2.00 mm, ML −1.90 mm, and DV −2.00 mm for dCA3 recording. After the procedure, all animals were allowed to recover for a week in their home cages.

### Elevated-Plus Maze Test

The elevated plus maze (EPM) test was performed at least one week after the mice recovered from the implantation surgery. The EPM consisted of 2 opposing closed arms (sidewalls, 30 × 5 × 25 cm) and 2 opposing open arms (open platforms, 30 × 5 cm) connected by a central stage (5 × 5 cm). The EPM was set 1 m from the floor. Before each trial, all arms and center stages were cleaned with 20% alcohol. The mice were placed individually on the central platform and allowed to explore for 10 min. For the chemogenetic experiments, the designer receptor ligand clozapine-N-oxide (CNO) was dissolved in 100% saline at a concentration of 0.10 mg/mL and administered intraperitoneally at a dose of 1 mg/kg 30 min before the commencement of behavioral testing. For the optogenetic experiments, blue light at a wavelength of 473 nm was delivered at a power of 10 mW in cycling stimulation mode (5 ms pulses at 20 Hz, 5 min off, 5 min on) *via* an optical fiber.

### Electrophysiological Recording

Electrophysiological signals were recorded using a 64-channel neural acquisition processor (Plexon, Dallas, TX, United States). Neural electrophysiological data acquired in this study were sampled at 40 kHz and bandpass filtered in the range of 300–5000 Hz. Synchronized mouse behavior was recorded using a digital video camera (Plexon, Dallas, TX, United States).

### Data Analysis

Data analyses were performed using Offline Sorter (Offline sorter application version 4.6.0), NeuroExplorer (NeuroExplorer version 5.310), and custom software written in MATLAB. Multi-unit recordings were high-pass filtered (300 Hz) with a Bessel filter for the detection of spikes. Individual spikes were detected by setting a threshold at −5 × standard deviations (SD), and spike waveforms were measured within a 1400 μs time window beginning 300 μs before threshold crossing. Single units were isolated using the first three principal components. To characterize putative GABAergic and glutaminergic neurons, two features of the extracellular waveform, the peak-to-peak time, and the half-width of the spikes, were calculated. Neurons with narrow peaks were regarded as putative GABAergic neurons, whereas neurons with wide peaks were regarded as putative glutamatergic neurons (Courtin et al., 2014; Lu et al., 2016). For the peri-event raster plot, RA-induced neural activity was calculated from 9 s segments of continuous neural recordings (from 6 s before to 3 s after RA onset) using a *z*-score transformation (bin = 0.5 s). *Z*-score values were calculated by subtracting the average baseline firing rate over the 6 s preceding RA onset and dividing by the baseline SD.

### Transcriptomic Sequencing

All animals recruited in this cohort arrived at our facility at the same time and completed the experimental procedures, including KA injection and sample collection, on the same day (Supplementary Table 1). Both wild type (WT) and TLE mice were euthanized in the same time window (3–4 p.m., 20-week-old, male) with a lethal dose of pentobarbital. Brains were extracted from the skull, and the hippocampus and lateral septum tissues were dissected, collected on ice, and frozen quickly. Total RNA from the hippocampus and lateral septum of the WT and TLE mice was utilized for library construction for sequencing by using the Illumina whole transcriptome RNA sequencer. All experimental protocols and transcriptome analyses were performed by trained technicians (Supplementary Figure 3).

All sequencing adapters were removed by running the Cutadapt. Quality control on FastQ files was checked by the FastQC application. All reads were aligned to UCSC genome browser (Mus musculus NCBIM37) by using HISAT2. We used HTSeq statistics to compare read count values for each gene as the original expression of the gene. Read counts are positively correlated with the true level of gene expression, as well as the length and sequencing depth of the gene. To make the level of gene expression comparable between different genes and samples, we used fragments per kilo bases per million fragments (FPKM) to standardize the expression (Normalization). We used DESeq to analyze the differences in gene expression, and the screening of the differentially expressed gene conditions was: expression difference multiples |log2FoldChange| >1, the significance *P*-value < 0.05. The R language ggplots2 software package was used to map the volcano of the differentially expressed genes. The volcanic map shows the distribution of genes, multiple gene expression differences, and significant results; under normal circumstances, the difference in gene distribution should be roughly symmetrical, the left side is Case compared to Control down gene, and the right side is compared to Case Control up gene. Genes that had no reads across all samples were discarded by the DESeq analysis.

### Histology

Briefly, the mice were perfused transcardially with 0.1 mol/L phosphate buffered saline (PBS) followed by 4% paraformaldehyde in 0.1 mol/L PBS, and the brains were removed and fixed at 4°C overnight, transferred to 30% sucrose in 0.1 M PBS 3 days, and then stored at −80°C. For immunostaining, 35 μm sections were cut using a cryostat (CM1950, Leica, Wetzlar, Germany). Subsequently, neurons were incubated overnight at 4°C with primary antibodies against somatostatin (Millipore, ab5494, 1:100, Darmstadt, Germany), neurotensin (Abcam, ab233107, 1:250, Cambridge, England), VIP (Abcam, ab272726, 1:250, Cambridge, England), and CCK (ImmunoStar, 20078, 1:250, Hudson, WI, United States) diluted in PBS containing 10% normal goat serum based on the antibody used. After extensive washing with PBS, secondary antibodies were added for 1 h at room temperature. Nuclei were counterstained with DAPI. Subsequently, the coverslips were washed with PBS and mounted onto glass slides with Southern Biotech fluorescence mounting medium.

### Microscopy and Quantitative Image Analysis

Fluorescence images were obtained using an Olympus VS120 microscope with UPLSAPO 10X and UPLSAPO 20X. Images containing 4–5 sections were used for the analysis of fluorescence intensities, counting of neurons, or the number of fluorescent spots in the case of *in situ* proximity ligation assay (PLA). The laser intensities and detector gain were adjusted such that all signals were below saturation. Images were analyzed using the Olympus CellSens Dimension Desktop 1.18.

### Statistics

Data were analyzed using two-tailed two-sample *t*-tests with the Origin software. All data are presented as the mean ± SD. Significance levels are indicated as follows: * *p* < 0.05, ^**^ *p* < 0.01, and ^***^ *p* < 0.001. The statistical details are shown in the respective figure legends.

## Results

### Heightened Approach-Risk Assessment Behavior of Kainic Acid-Induced Temporal Lobe Epilepsy Mice in Elevated Plus Maze

**FIGURE 1:**
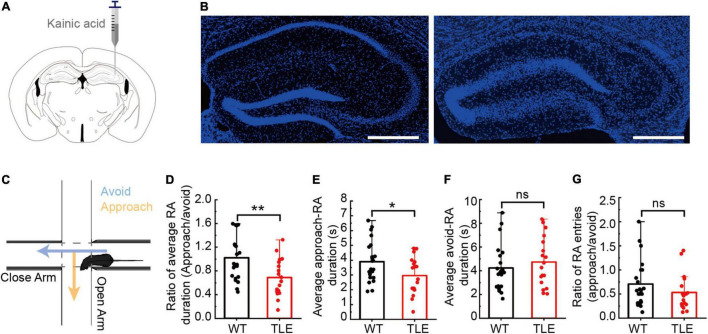
Kainic acid-induced TLE mice showed impairments in the hippocampal cytoarchitecture and RA behavior. **(A)** Schematic diagram of intrahippocampal kainic acid injection in C57/BL6J mice. **(B)** Comparison of hippocampal subfields between WT and TLE mice. Scale bar, 500 μm. **(C)** Schematic diagram of elevated plus maze test and a typical RA. **(D)** Ratio between average of approach-RA duration and average of avoid-RA duration. **(E)** Average of approach-RA duration. **(F)** Average of avoid-RA duration. **(G)** Ratio between approach-RA and avoid-RA entries (WT, *n* = 22; TLE, *n* = 18; ns is no significant difference; error bars represent s.d; *p < 0.05; **p < 0.01, *t* test).

To obtain a stable mouse model of chronic temporal lobe epilepsy, we used an intrahippocampal kainic acid injection strategy (Figure 1A). In the KA-injected group, the CA1 sector exhibited severe neuronal loss, whereas, in the control group, layers of the CA1 and CA3 sectors and the dentate gyrus appeared intact (Figure 1B and Supplementary Figure 2, *p* < 0.0001). Stable hippocampal sclerosis in TLE mice demonstrated the efficiency of the KA-induced TLE mouse model. To investigate whether the impairment of the hippocampus cast a shadow on the RA, we used EPM and focused on a typical RA behavior with a forthcoming approach or avoidance (Figure 1C). Here, we defined approach-RA as that with a forthcoming approach to the open arm, and avoid-RA that with a forthcoming avoidance of the open arm. Compared with WT mice, the TLE mice showed a significant decrease in approach-RA duration (WT: 3.90 ± 1.37, TLE: 2.96 ± 1.26, *p* = 0.0307, Figure 1E) but not avoid-RA duration (WT: 4.23 ± 1.93, TLE: 4.74 ± 2.08, *p* = 0.4341, Figure 1F). Moreover, the ratio between approach-RA duration and avoid-RA duration even altered more significantly (WT: 1.02 ± 0.38, TLE: 0.69 ± 0.31, *p* = 0.0051, Figure 1D). The ratio between entries of approach-RA and avoid-RA did not significantly differ (WT: 0.64 ± 0.59, TLE: 0.71 ± 0.49, *p* = 0.7042, Figure 1G). These results indicated that the TLE mice spent less time in approach-RA and decreased latency from the close arm to the open arm, which led to more vulnerable animals. Taken together, intrahippocampal KA injection-induced TLE mice exhibited neuronal degeneration, DG dispersion, and reorganization of the hippocampal structure, and significantly destroyed RA, facing potential threat.

### Excitation and Inhibition Imbalance of Dorsal Dentate Gyrus and CA3-dLS in Approach-Risk Assessment

**FIGURE 2:**
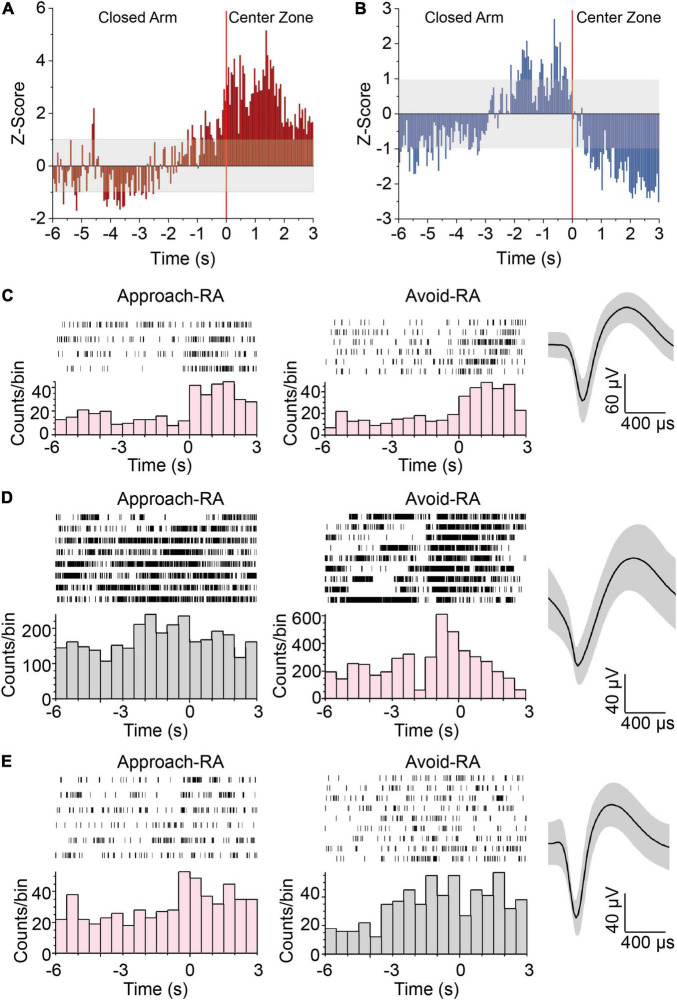
Neurons in HPC responding to approach-RA or avoid-RA selectively. **(A,B)** *Z*-scored peri-RA time histograms aligned with onset of RA. **(A)** *n* = 57 cells from 5 mice. **(B)** *n* = 60 cells from 5 mice. **(C)** Representative raster plots and waveforms of dDG/CA3 neurons positively responsive to RA. **(D)** Representative raster plots and waveforms of dDG/CA3 neurons positively responsive to avoid RA. **(E)** Representative raster plots and waveforms of dDG/CA3 neurons positively responsive to approach RA. Each row in the raster represents a single trial of RA behavior. Averaged spike waveforms of the representative neuron were provided at the left panels. Bin = 0.5 s.

Considered patterns of hippocampal oscillation predicting approach-RA and avoid-RA has been reported (Jacinto et al., 2016), to confirm whether the hippocampal neurons can also predict approach-RA and avoid-RA, we recorded and analyzed the electrophysiological signals in dDG/CA3 and dLS during EPM. We found that the amounts of glutamatergic and GABAergic neurons were activated by RA (Figure 2A), most of which were positively responsive to both approach-RA and avoid-RA (Figure 2C). However, there are subpopulations of RA-activated neurons that preferentially respond to either approach-RA or avoid-RA (Figures 2D,E). In this study, we found that some of RA-inhibited neurons exhibited a phasic increase of firing rate before RA (in close arm, duration, ∼2–3 s) and subsequently a decrease of firing rate during RA (in center zone) (Figure 2B and Supplementary Figure 1). Thus, we considered the RA-inhibited neurons as another behavior-related neurons, and did not discuss in current study. The selectively responsive neurons may contribute to the changed pattern of theta oscillations to predict the forthcoming approach or avoid.

**FIGURE 3:**
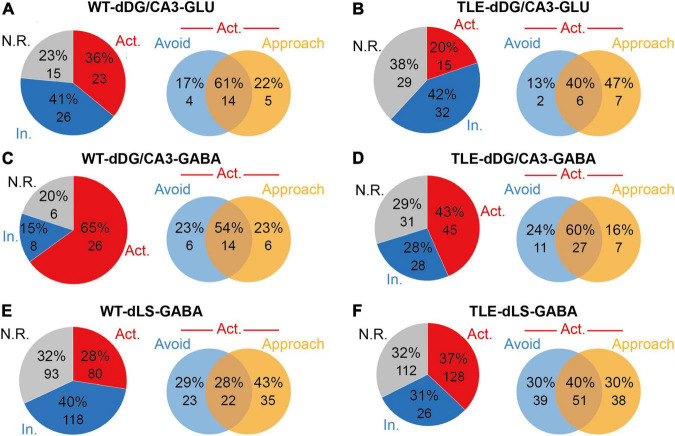
Electrophysiological analysis showed E/I imbalance in TLE mice. **(A)** Neuronal response types of putative glutamatergic neurons to RA in dDG/CA3 of WT mice [Red, excited (Act.); blue, inhibited (In.); gray, no response (N.R.). *n* = 64 cells from 5 mice]. Venn diagram shows the distribution of approached RA-excited only, avoid RA-excited only or dual-excited putative glutamatergic neurons in dDG/CA3 of WT mice at RA. **(B)** Neuronal response types of putative glutamatergic neurons to RA in dDG/CA3 of TLE mice (Red, excited; blue, inhibited; gray, no response; *n* = 76 cells from 5 mice). Venn diagram shows the distribution of approached RA-excited only, avoid RA-excited only or dual-excited putative glutamatergic neurons in dDG/CA3 of TLE mice at RA. **(C)** Neuronal response types of putative GABAergic neurons to RA in dDG/CA3 of WT mice (Red, excited; blue, inhibited; gray, no response; *n* = 40 cells from 17 mice). Venn diagram shows the distribution of approached RA-excited only, avoid RA-excited only or dual-excited putative glutamatergic neurons in dDG/CA3 of WT mice at RA. **(D)** Neuronal response types of putative GABAergic neurons to RA in dDG/CA3 of TLE mice (Red, excited; blue, inhibited; gray, no response; *n* = 104 cells from 15 mice). Venn diagram shows the distribution of approached RA-excited only, avoid RA-excited only or dual-excited putative glutamatergic neurons in dDG/CA3 of TLE mice at RA. **(E)** Neuronal response types of putative GABAergic neurons to RA in dDG/CA3 of WT mice (Red, excited; blue, inhibited; gray, no response; *n* = 291 cells from mice). Venn diagram shows the distribution of approached RA-excited only, avoid RA-excited only or dual-excited putative glutamatergic neurons in dLS of WT mice at RA. **(F)** Neuronal response types of putative GABAergic neurons to RA in dDG/CA3 of TLE mice (Red, excited; blue, inhibited; gray, no response; *n* = 346 cells from mice). Venn diagram shows the distribution of approached RA-excited only, avoid RA-excited only or dual-excited putative glutamatergic neurons in dLS of TLE mice at RA.

To elucidate the role of responsive neurons in the impaired RA of TLE mice, we examined all neurons recorded from unbiased populations of dDG/CA3 or dLS in WT and TLE mice. During RA, the proportion of inhibited dDG/CA3 putative glutamatergic neurons showed no obvious change between TLE (41%) and WT (42%) mice, whereas the proportion of excited dDG/CA3 putative glutamatergic neurons decreased by 16% in TLE mice (WT, 36%; TLE, 20%) (Figures 3A,B). The proportion of excited putative GABAergic neurons in the HPC was 22% lower in the TLE group than the WT group (WT, 65%; TLE, 43%) (Figures 3C,D), and RA was found to inhibit putative GABAergic neurons in the HPC by 12% (WT, 15%; TLE, 27%). Therefore, both types of RA-excited HPC neurons were decreased in TLE mice, which may play a role in the impaired RA of epileptic mice.

Of note, when compared with the avoid-RA selectively responsive neurons, the approach-RA selectively excited dDG/CA3 putative glutamatergic neurons increased (WT, 22%; TLE, 47%), for which the percentage was defined as either approach-RA selectively excited neurons divided by all RA-excited neurons, or avoid-RA selectively excited neurons divided by all excited neurons (Figures 3A,B). On the other hand, the proportion of approach-RA selectively excited dDG/CA3 putative GABAergic neurons showed a slight unexpected decrease (WT, 23%; TLE, 16%) while the proportion for avoid-RA remained unchanged (Figures 3C,D). Taken together, in the TLE-impaired approach-RA, the E/I balance changed markedly, and the approach-RA preferentially activated glutamatergic neurons increased, whereas the approach-RA preferentially activated GABAergic neurons decreased.

Similarly, the proportion of RA-excited putative GABAergic neurons in the LS was slightly increased (WT, 28%; TLE, 37%), while the approach-RA selectively excited putative GABAergic neurons in the LS (WT, 43%; TLE, 30%) decreased more than those for avoid-RA (WT, 29%; TLE, 30%) (Figures 3E,F). Taken together, these findings suggest that in TLE mice, approach-RA selectively activated neurons were changed more significantly in both the dDG/CA3 and the dLS. In particular, approach-RA selectively responsive neurons displayed an E/I imbalance in the dDG/CA3. Therefore, the RA-recruited neuronal network in the dDG/CA3-dLS areas of the brain may be vulnerable to epilepsy due to degeneration and reorganization.

### Translational Signatures Imply Apoptosis, Impairment of Neurogenesis, and Migration

**FIGURE 4:**
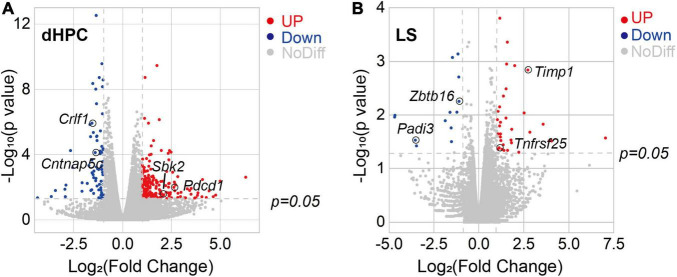
Kainic acid-induced TLE mice showed transcriptome alterations in the hippocampus and lateral septum. **(A)** Volcano plot of relative RNA expression in DG/CA3 from TLE mice as compared to WT mice. **(B)** Volcano plot of relative RNA expression in dLS from TLE mice as compared to WT mice [Genes in upper left and right quadrants are significantly differentially expressed. Red dots, up-regulation (dHPC, 65 genes; LS, 34 genes), blue dots, down-regulation (dHPC, 224 genes; LS, 11 genes). WT, *n* = 4 mice; TLE, *n* = 6 mice. *p* < 0.05].

To investigate the causes of impaired dysfunction of hippocampus and lateral septum neurons at the molecular level, we used RNA-seq technology to characterize the transcriptome of dHPC and LS of TLE mice (Supplementary Table 1). The normalized expression analysis of all samples from both the TLE and WT groups was used to visualize the differences in expression of all detected genes in both dHPC and LS. As expected, a large number of genes were expressed at higher levels in the dHPCs of TLE mice (Figure 4A). Similarly, we found differentially expressed genes in the LS (Figure 4B). To further explore the link between the impaired approach-RA and the change in transcription in TLE mice, we examined gene expression for Gene Ontology terms. Most of the genes examined showed similar expression between TLE and WT mice. However, several genes were upregulated or downregulated in neurogenesis, migration, and apoptosis in dHPC, as well as migration and apoptosis in LS (Table 1).

**TABLE 1:** Representative dysregulated genes in HPC and LS of TLE mice.

Brain region	ID	Chromosome	Name	Fold change	Description
HPC	ENSMUSG00000009900	11	Wnt3a	−, 0	wingless-type MMTV integration site family, member 3A
	ENSMUSG00000054409	15	Crlf1	−, 0.336	cytokine receptor-like factor 1
	ENSMUSG00000041911	2	Dlx1	−, 0.494	distal-less homeobox 1
	ENSMUSG00000021676	13	Iqgap2	−, 0.313	IQ motif containing GTPase activating protein 2
	ENSMUSG00000038048	17	Cntnap5c	−, 0.377	contactin associated protein-like 5C
	ENSMUSG00000069310	13	H3c3	+, infinite	H3 clustered histone 3
	ENSMUSG00000043557	17	Mdga1	−,0.488	MAM domain containing glycosylphosphatidylinositol anchor 1
	ENSMUSG00000026285	1	Pdcd1	+, 6.157	programmed cell death 1
	ENSMUSG00000030433	7	Sbk2	+, 5.395	SH3-binding domain kinase family, member 2
LS	ENSMUSG00000026343	1	Gpr39	+, infinite	G protein−coupled receptor 39
	ENSMUSG00000024793	4	Tnfrsf25	+, 2.191	tumor necrosis factor receptor superfamily, member 25
	ENSMUSG00000066687	9	Zbtb16	−, 0.464	zinc finger and BTB domain containing 16
	ENSMUSG00000001131	X	Timp1	+, 6.603	tissue inhibitor of metalloproteinase 1
	ENSMUSG00000025328	4	Padi3	−, 0.087	peptidyl arginine deiminase, type III

For example, Wnt3a acts through canonical Wnt signaling to drive embryonic development, stem cell differentiation, and promote neural circuit formation, as well as neurogenesis in the hippocampus (Rash et al., 2013; Harada et al., 2019). Here, Wnt3a expression was not detected in the TLE hippocampus, suggesting that neurogenesis was disrupted. In addition, Crlf1 is also considered to play a crucial role during neuronal development (Johnson et al., 2010) and Dlx1 was found to participate in the differentiation of interneurons (He et al., 2001; Cobos et al., 2006), such as bipolar cells in the developing retina; thus, their downregulation may be related to neurogenesis dysfunction, especially E/I imbalance. Moreover, we found through the immunostaining of BrdU+ neurons in the HPC that the number of newborn neurons was significantly decreased in TLE mice (Supplementary Figure 4), which confirmed that there was an impairment of neurogenesis due to TLE. In short, downregulation of neurogenesis may result in an E/I imbalance and subsequently contribute to impaired RA.

The Iqgap2 gene encodes a protein that interacts with components of the cytoskeleton, which is related to cell–cell adhesion, cell migration, and cell signaling, as well as crosstalk with the Wnt pathway (Smith et al., 2015). The Cntnap5c gene is a contactin-associated protein that is important for cell adhesion and intercellular communication in the nervous system (Hirata et al., 2016). Mdga1 encodes a glycosylphosphatidylinositol (GPI)-anchored cell surface glycoprotein that is suggested to play a role in neuronal migration, axon outgrowth, and axon-target recognition (Litwack et al., 2004). Furthermore, the Mdga1 and Cntnap5c genes were found to be crucial for balancing excitatory and inhibitory neurotransmission (Elegheert et al., 2017; Tong et al., 2019). Taken together, the downregulation of Iqgap2, Cntnap5c, and Mdga1 confirmed that in TLE, there is DG dispersion, neuronal reorganization, as well as neuronal disconnection and E/I imbalance.

Pdcd1 encodes programmed cell death protein 1, which indicates cell death of the nervous tissue following chronic injury (Pu et al., 2018). Tnfrsf25 gene production is known as death receptor 3, which is involved in the regulation of apoptosis, and is increased in slowly expanding lesions (Jackle et al., 2020). Timp1 mRNA was found after neuronal injury, and the Timp1 gene product was considered to have an anti-apoptotic function (Huang et al., 2011). Zbtb16 is involved in cell cycle progression and is used as a boundary cell marker (Takahashi and Osumi, 2011). Moreover, Zbtb16 was shown to be downregulated after nerve injury (Zhang et al., 2019). Thus, the decrease in Pdcd1 gene transcription confirmed neuronal loss in the TLE hippocampus; both Timp1 and Tnfrsf25 increase, Zbtb16 decrease may also be related to dLS neuron apoptosis and neuronal dysfunction.

Notably, Gpr39 was reported to be related to neurotensin receptors (NTSR) (Popovics and Stewart, 2011). In our study, we found that Gpr39 transcription was increased. Taken together, the transcriptome analysis results revealed that gene dysfunction contributed to neuronal loss, neural network reorganization, and downregulation of neurogenesis in TLE dHPC and may cause E/I imbalance and risk assessment impairments. Interestingly, dLS also exhibited the dysregulation of related genes, indicating that intrahippocampal kainic acid injection can result in dLS-related circuit reorganization.

### Degeneration of Dorsal Dentate Gyrus and CA3 Somatostatin-Positive Neurons and dLS Cholecystokinin-Positive Neurons May Contribute to Excitation and Inhibition Imbalance

**FIGURE 5:**
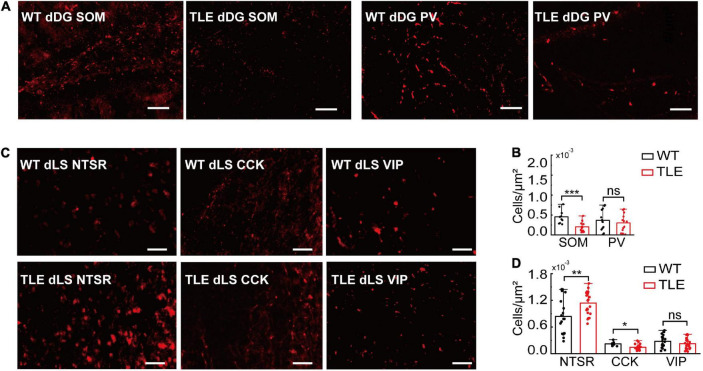
Comparison of GABAergic neurons subtypes in WT vs. TLE. **(A)** Representative images of immunostaining of PV+ and SOM+ neurons in dDG of WT and TLE mice. Scale bar, 200 μm. **(B)** The densities of SOM+ and PV+ neurons in dDG/CA3 (SOM+: WT, *n* = 6 mice, TLE, *n* = 6 mice; PV+: WT, *n* = 6 mice, TLE, *n* = 5 mice). **(C)** Representative images of immunostaining of NTSR+, CCK+, and VIP+ neurons in dLS of WT and TLE mice. Scale bar, 200 μm. **(D)** The densities of NTSR+, CCK+, and VIP+ neurons in dLS (NTSR+: WT, *n* = 11 mice, TLE, *n* = 10 mice; CCK+: WT, *n* = 6 mice, TLE, *n* = 9 mice, VIP+: WT, *n* = 11 mice, TLE, *n* = 6 mice) (Error bars represent s.d; *p < 0.05; **p < 0.01, ***p < 0.001, *t-*test).

To examine the effect of KA injection on hippocampal and lateral septal GABAergic neurons, we performed immunostaining for SOM+ and PV+ in dDG/CA3 and NTSR+, CCK+, and VIP+ in the dLS. The SOM+ neurons in dDG/CA3 were significantly decreased in TLE mice compared with WT mice, whereas PV+ neurons showed no significant change (SOM+, *p* = 0.0002626; Figures 5A,B). However, morphological changes in PV+ neurons were observed, indicating the alteration of PV+ neurons function (Supplementary Figure 5). The loss of SOM+ neurons may contribute to E/I imbalance in the HPC and impaired RA. In dLS, NTSR+ neurons were significantly increased, in accordance with transcription-related results, whereas CCK+ neurons decreased (NTSR+, *p* = 0.00912, CCK+, *p* = 0.0042; Figures 5C,D), which may be due to decreased GABAergic neurons in the dLS and impaired RA. Therefore, GABAergic neurons in both the dHPC and dLS were altered in TLE mice, and different subtypes showed different vulnerabilities. These GABAergic neurons may have a complicated involvement in the E/I imbalance and RA impairment.

### Manipulation of Dorsal Dentate Gyrus and CA3 CaMKII+ or Somatostatin-Positive Neurons Is Sufficient to Impair Risk Assessment

**FIGURE 6:**
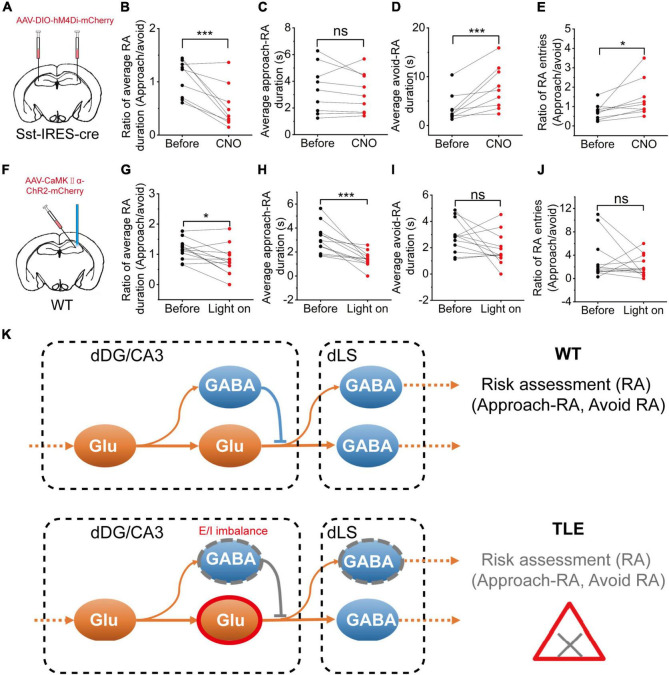
Chemogenetic inhibition of hippocampal SOM+ neurons or optogenetic activation of CaMKII+ neurons impaired the RA of mice. **(A)** Diagram showing the chemogenetic inhibition of CA3 SOM+ neurons; **(B)** Ratio between the mean approach- and avoid-RA durations; **(C)** Average approach-RA duration; **(D)** Average avoid-RA duration; **(E)** Ratio between approach- and avoid-RA entries (before CNO, *n* = 9; CNO, *n* = 9; error bars represent standard deviation (s.d.); *p < 0.05; ***p < 0.001, paired *t*-test); **(F)** Diagram of the experimental setup for optogenetic stimulations; **(G–J)** Optogenetic activation of hippocampal glutamatergic neurons; **(G)** Ratio between the mean approach- and avoid-RA durations; **(H)** Average approach-RA duration; **(I)** Average avoid-RA duration; **(J)** Ratio between approach- and avoid-RA entries (before light, *n* = 12; light on, *n* = 12; error bars represent standard deviation (s.d.); ***p < 0.001, paired *t*-test); and **(K)** A supposed model of neural E/I imbalance mechanism in impairment of approach-RA in TLE. ns, no significant difference.

Our electrophysiological and histological data indicated that SOM+ neurons in the HPC were decreased in KA-induced TLE mice, which may contribute to RA impairment. Therefore, we hypothesized that the direct inhibition of SOM+ neurons in the HPC *in vivo* would be sufficient to alter RA. To investigate this hypothesis, we used chemogenetic in combination with the EPM. To selectively inhibit SOM+ neurons in the HPC, we bilaterally expressed hM4Di in these neurons (Figure 6A). As compared to the group without clozapine-N-oxide (CNO) administration, the CNO administration group showed a significant decrease in the ratio of the average approach- and avoid-RA durations (Figure 6B). These results showed that the inhibition of dHPC SOM+ neurons led to an impairment in RA behavior. Furthermore, the inhibition of SOM+ neurons caused an increase in the ratio of approach- to avoid-RA entries, suggesting that after RA, the animals have a higher probability of exploring the open arm than the closed arm (Figure 6E). Taken together, these results imply that these animals are more likely susceptible to danger.

Interestingly, the inhibition of SOM+ neurons in the HPC led to an increased average duration of avoid-RA (Figure 6D), while did not affect average duration of approach-RA (Figure 6C). It is widely accepted that closed arms are relatively safe compared with open arms. To further investigate the meaning of the average duration of avoid-RA, we noted that both the entries and total duration of avoid-RA had decreased consistently (Supplementary Figures 6A–D), suggesting that the CNO administration group suppressed the preference of the closed arm (safe zone). These results further substantiate the notion that selective inhibition of the SOM+ neurons in the HPC leads to RA impairment in mice after CNO administration.

To further investigate whether the activation of glutamatergic neurons in the HPC impairs RA, we selectively applied optogenetic activation to these neurons in the EPM (Figure 6F). The optogenetic activation showed no effect on the avoid-RA (Figure 6I and Supplementary Figures 6G,H), while the ratio of approach- and avoid-RA was no significant difference (Figures 6G,J). However, both of the average and total approach-RA duration were significantly decreased (Figure 6H and Supplementary Figures 6E,F). Taken together, the chemogenetic inhibition and optogenetic activation results, which were similar to the TLE mice, confirmed that the E/I imbalance of HPC contributed to impairment of RA behavior. Moreover, the heterogeneous loss of neurons, which may be modulated by dysregulation of apoptosis, neurogenesis and migration, was related to the E/I imbalance in KA-induced TLE mice.

## Discussion

The current study confirms and extends previous studies on the epilepsy-induced impairment of RA and the future exploratory outcome, in which E/I imbalance in dDG/CA3 and alteration of dLS contributed. Animal behavior in the EPM can be quantified as anxiety, exploration, and RA. Recent studies have suggested that animals with epilepsy have impaired RA (Kubova et al., 2004; O’Loughlin et al., 2014), and we found that the impaired RA led to epileptic mice preferentially approaching potential threats. Previous studies have shown that the hippocampus and its broad of downstream participate in regulating behavior in EPM (Lee et al., 2019; Besnard et al., 2020; Xia and Kheirbek, 2020; Botterill et al., 2021; Dong et al., 2021; Wang et al., 2021), in which RA behavior plays a key role in future outcomes (Jacinto et al., 2016; Motta et al., 2017; Blanchard, 2018; McNaughton and Corr, 2018). In this study, we confirmed that dDG/CA3 neurons are involved in RA behavior. Moreover, subpopulations of activated neurons preferentially responded to either approach-RA or avoid-RA. In epileptic mice, the impaired approach-RA was attributed to the E/I imbalance of dDG/CA3. In addition, a decrease in the approach-RA-specifically activated GABAergic neurons may also contribute toward it.

Risk assessment is considered a core component of the defense survival system, especially when dealing with ambiguous or mild threats (Gross and Canteras, 2012; Blanchard, 2018). Therefore, an animal’s behavior in the central stage of the EPM is considered to be RA (Rodgers and Dalvi, 1997), which can help an animal detect potential threats, while the animal’s internal state, such as anxiety, can also affect its RA behavior (Blanchard et al., 2011). Previous studies have shown that hippocampal neuronal activity changes as the experimental subject moves to different regions of the EPM (open or closed arms), focusing on anxiety behavior (Adhikari et al., 2011; Felix-Ortiz et al., 2013; Padilla-Coreano et al., 2016; Lee et al., 2019). Moreover, hippocampal neurons also participate in RA behavior (Motta et al., 2017; McNaughton and Corr, 2018), and hippocampal theta oscillations are recruited in RA, and their pattern can predict future exploratory outcomes, including approach and avoidance to the open arms (Jacinto et al., 2016. Here, we directly demonstrate, at the neuronal level, that among the RA-activated dDG/CA3 neurons, ∼22% of glutamatergic and ∼23% of GABAergic neurons are preferentially responsive to approach-RA, while ∼17% of glutamatergic and ∼23% of GABAergic neurons are preferentially responsive to avoid-RA. Notably, several studies have dissected the RA mechanism focusing on the ventral hippocampus because it is more involved in emotions, such as anxiety (Felix-Ortiz et al., 2013). As the RA stands at the interface of cognition and emotion (Blanchard, 2018), we determined that the dorsal hippocampal dDG/CA3 also processed the RA information. In the future, depending on the activity of the dDG/CA3 neurons, we may predict the following behavioral outcomes. In addition, the dLS, as the key node between the hippocampus and subcortical regions, is also engaged in RA (Motta et al., 2017). We further discovered that dLS neurons could specifically activate either approach-RA or avoid-RA, similar to dDG/CA3 neurons.

Epileptic animals were found to have reduced RA, which may be related to the impairment of fear and anxiety (O’Loughlin et al., 2014). Here, we evaluated RA and analyzed the approach-RA and avoid-RA behaviors, and more accurately revealed that the duration of approach-RA in epileptic mice was reduced significantly, but not in the avoid-RA. Thus, the epileptic mice are specifically impaired with approach-RA, so that they may have a tendency to underestimate the potential threat. Notably, we observed that a few epileptic mice fell from the open arm in the EPM (these samples were excluded from our results), but no WT mice were found to fall. It has been reported that epileptic patients have a higher risk of premature mortality due to external causes (Fazel et al., 2013), and our results imply that the impairment of estimating the potential risk may contribute to it.

Recurrent seizures lead to significant structural and functional disability of the involved brain regions, including dysconnectivity of the cortex and hippocampus (Fadaie et al., 2021). As a result, it is widely accepted that this can lead to brain disorders with broad cognitive impairments (Fadaie et al., 2021). Seizures in TLE disable the function of the hippocampus and its downstream brain regions, including the LS, entorhinal cortex, and broad cortex areas (Motelow et al., 2015; Lu et al., 2016). KA-induced TLE rodents show obvious hippocampal apoptosis, similar to epileptic patients. Moreover, the loss of GABAergic neurons is even more severe (Wyeth et al., 2020). In the present study, we found that an increase in the dDG/CA3 glutamatergic neurons and a decrease in dDG/CA3 and dLS GABAergic neurons in the HPC of the mice with TLE resulted in the impairment of approach-RA, but not avoid-RA. Interestingly, our results also suggested that approach-RA-activated dDG/CA3 glutamatergic neurons increased in accordance with hyperexcitation of glutamatergic neurons in seizures in a previous study (Krook-Magnuson et al., 2013). In summary, our results suggest that in recurrent seizures, the E/I imbalance of the hippocampus induces inhomogeneous neuronal lesions and induces dysfunction of RA.

In TLE, different types of GABAergic neurons suffer heterogeneous damage, such as SOM+ neurons optionally located in the hilus, which are more vulnerable than PV+ neurons in HPC both in animals and humans (Swartz et al., 2006; Marx et al., 2013). In this study, we confirmed that the SOM+ neurons were significantly degenerated compared with PV+ neurons. The VIP+ GABAergic neurons were found to preferentially innervate inhibitory interneurons, such as CCK+, SOM+, and PV+ neurons, highlighting their central role in disinhibitory modulation in HPC (Kullander and Topolnik, 2021). In addition, the VIP+ neurons were found to code information of the open and closed arms in the EPM (Lee et al., 2019). CCK+ neurons are involved in relevant behavioral functions, such as memory, cognition, anxiety, and reward, by regulating their cognitive components (Ballaz and Bourin, 2021). Herein, we found that the number of dLS CCK+ neurons in TLE was significantly decreased, but not in NTSR+ or VIP+ neurons. Direct manipulation of glutamatergic or GABAergic neurons in the HPC results in the impairment of RA, and may also can the animal to underestimate a potential threat. As such, we hypothesized that the activation of dDG/CA3 glutamatergic neurons and the loss of dDG/CA3 SOM+ neurons contributed to E/I imbalance and impairment of RA in mice with TLE (Figure 6K).

It is known that epilepsy not only induces hippocampal neurodegeneration, hyper synchronicity, and E/I imbalance, but also leads to the formation of abnormal hippocampal circuitry reorganization, such as granule cell dispersion. The hippocampus has a complex brain structure with a special regular organization. Based on this, the hippocampus is a super plastic structure, which is critical for a broad function of cognition, emotion, and motivation, while it is vulnerable to damage, especially in epilepsy. Thus, the TLE-induced reorganization and disconnection of the hippocampus may cause approach-RA, even though approach-RA-related neurons did not degenerate. In addition, we found that hippocampal PV+ neurons have altered morphology, so that the disconnection of PV+ neurons may contribute to impaired approach-RA. In the DG, axons of the entorhinal cortex project onto dentate granule cells (glutamatergic neurons) and onto GABAergic interneurons (including PV+ and SOM+ cells) as well as mossy cells (glutamatergic neurons) in the hilus. Furthermore, the DG/hilus are considered as a gate, in which hilar mossy cells play a critical role and are vulnerable to excitotoxicity in TLE (Goldberg and Coulter, 2013). Thus, TLE may decrease the inhibition of survival of GABAergic neurons. In summary, the reorganization of the DG-CA3 network, both the rearrangement of mossy fiber sprouting and PV+ neurons, may also contribute to the E/I imbalance, then selectively damage the approach-RA function.

Specifically, adult hippocampal neurogenesis has been discovered, and these newborn neurons are important for hippocampal functions (Anacker and Hen, 2017). With the discovery of adult neurogenesis and its understanding, the role of neurogenesis in epilepsy needs to be addressed. After seizures, dentate neurogenesis increase has been reported (Goldberg and Coulter, 2013), whereas others have found that neurogenesis is disrupted (Lauren et al., 2013; Marucci et al., 2013). Our results suggest that neurogenesis in the epileptic hippocampus was abnormal, as evidenced by the downregulation of related transcriptional signals, such as Wnt3a, Crlf1, and Dlx1. In accordance with a study on chronic epilepsy, we suppose that an increase in neurogenesis may occur in the acute phase, but a decrease in neurogenesis occurs in the chronic phase (Huang et al., 2015). Furthermore, newborn neurons were thought to inhibit mature granule cells, as silencing them affected the animal’s stress-related behaviors (Anacker et al., 2018). Future studies should investigate whether the impairment of neurogenesis affects RA in TLE.

In conclusion, we found a decrease in the capacity of RA in epileptic animals, which may lead epileptic individuals to underestimate the potential threat and ultimately cause injury. There were subpopulations of neurons in both the hippocampus and the lateral septum that could specifically actively respond to approach-RA, while maintaining a certain balance between excitatory and inhibition. However, in epileptic animals, these neurons are altered, and the E/I balance is disrupted. Transcriptome and immunohistochemical staining showed that neurons in the hippocampus and LS were lost, and neural networks were reorganized. In particular, dDG/CA3 SOM+ neurons and dLS CCK+ neurons are selectively vulnerable to damage in the TLE, whereas the dCA3/DG glutamatergic neurons are considered to be overexcited (Goldberg and Coulter, 2013). In the present study, we demonstrated that when combined with the manipulation of CaMKII+ and SOM+ neurons, an E/I imbalance of the dCA3/DG-dLS circuit contributes to the impairment of RA behavior and alternates the future exploratory outcome during epileptic individuals facing potential threat. The understanding of epileptic-induced impaired risk assessment at multiple levels would shed light on the understanding of E/I balance in brain function and provide a potential paradigm for related studies.

## Data Availability Statement

The raw data supporting the conclusions of this article will be made available by the authors, without undue reservation.

## Ethics Statement

The animal study was reviewed and approved by Shenzhen Institute of Advanced Technology, Chinese Academy of Sciences.

## Author Contributions

CZ, YL, CS, and YC were responsible for the conception of the study, design of experiments, and interpretation of the data. CS, YC, JH, and LW acquired the data. CZ, CS, YC, JH, PS, SH, and JL analyzed the data. CZ, CS, and YC wrote the manuscript. YL commented on this manuscript. All authors contributed to the manuscript and approved the submitted version.

## Conflict of Interest

The authors declare that the research was conducted in the absence of any commercial or financial relationships that could be construed as a potential conflict of interest.

## Publisher’s Note

All claims expressed in this article are solely those of the authors and do not necessarily represent those of their affiliated organizations, or those of the publisher, the editors and the reviewers. Any product that may be evaluated in this article, or claim that may be made by its manufacturer, is not guaranteed or endorsed by the publisher.

## Supplementary Material

Supplementary Figure 1 Neuronal classification and activity responses. **(A)** HPC neurons (*n* = 180 neurons from 5 mice) were classified by valley-to-peak time and spike half-width when the mice entered the food zone; blue, putative GABAergic neurons; red, putative glutamatergic neurons; **(B)** Quantified neuronal responses to approach-RA, where blue hollow rectangle = no response GABAergic neurons, red hollow rectangle = no response glutamatergic neurons, blue triangle = approach-RA excited GABAergic neurons, red triangle = approach-RA excited glutamatergic neurons, blue circle = approach-RA inhibited GABAergic neurons, and red circle = approach-RA inhibited glutamatergic neurons; and **(C)** Representative raster plots and waveforms of dDG/CA3 neurons responding negatively to RA, in which RA-inhibited neurons exhibited a phasic increase in the firing rate before RA (in close arm, duration, ∼2–3 s) and a subsequent decrease of the firing rate during RA (in center zone).

Supplementary Figure 2 The DAPI positive cells were reduced. The ratios were the fluorescence positive area (DAPI+ signal) divided area of ROI (region of interest) in dDG/CA3. WT, *n* = 22 slices from 6 mice, TLE, *n* = 27 slices from 6 mice. Error bars represent s.d. ^***^*p* < 0.001. Two-sample *t*-test.

Supplementary Figure 3 Overview of RNA sequencing flow diagram. Wild-type 6-week-old C57BL/6J male mice were obtained and housed for 2-week environmental acclimation. Half of the wild-type 8-week-old mice were subjected to KA-injection. All mice were housed to 20-week-old for sampling.

Supplementary Figure 4 Comparison of BrdU+ neurons in WT vs. TLE. **(A)** Representative images of immunostaining of BrdU+ neurons in dDG of WT and TLE mice. Scale bar, 50 μm. **(B)** The number of BrdU+ neurons in dDG (BrdU+: WT, *n* = 14 slices from 2 mice, TLE, *n* = 8 slices from 3 mice) (Error bars represent s.d; ^**^*p* < 0.01, two-sample *t* test).

Supplementary Figure 5 Morphological changes in PV+ neurons. Representative confocal images at 40× showed that the morphology of PV+ neurons cell body altered in dDG/CA3 of TLE mice. Scale bar, 20 μm.

Supplementary Figure 6 Chemogenetics inhibition of hippocampal SOM+ neurons or optogenetic activation of hippocampal glutamatergic neurons impaired the RA. **(A–D)** Chemogenetics inhibition of hippocampal SOM+ neurons. **(A)** The approach-RA duration. **(B)** The approach-RA entries. **(C)** The avoid-RA duration. **(D)** The avoid-RA entries (before CNO, *n* = 9; CNO administration, *n* = 9). **(E–H)** Optogenetic activation of hippocampal glutamatergic neurons. **(E)** The approach-RA duration. **(F)** The approach-RA entries. **(G)** The avoid-RA duration. **(H)** The avoid-RA entries (before light, *n* = 12; light on, *n* = 12) (ns is no significant difference; error bars represent s.d; **p* < 0.05; ^***^*p* < 0.001, paired-*t* test).

## Abbreviations

TLE: temporal lobe epilepsy
WT: wild type
RA: risk assessment
dHPC: dorsal hippocampus
HPC: hippocampus
EPM: elevated plus maze
E/I: excitation and inhibition
SOM+: somatostatin-positive
CCK+: cholecystokinin-positive
PV+: parvalbumin-positive
VIP+: vasoactive intestinal polypeptide positive
LS: lateral septum
HPC-LS: hippocampal-lateral section
dDG/CA3: dorsal dentate gyrus and CA3
ns: no significant
N.R.: no response.
