# Supplementary figures and images for: Dysfunction of the Hippocampal-Lateral Septal Circuit Impairs Risk Assessment in Epileptic Mice
Source: Front Mol Neurosci. 2022 Apr 29;15:828891. doi: 10.3389/fnmol.2022.828891 (PMC9103201; doi:10.3389/fnmol.2022.828891)

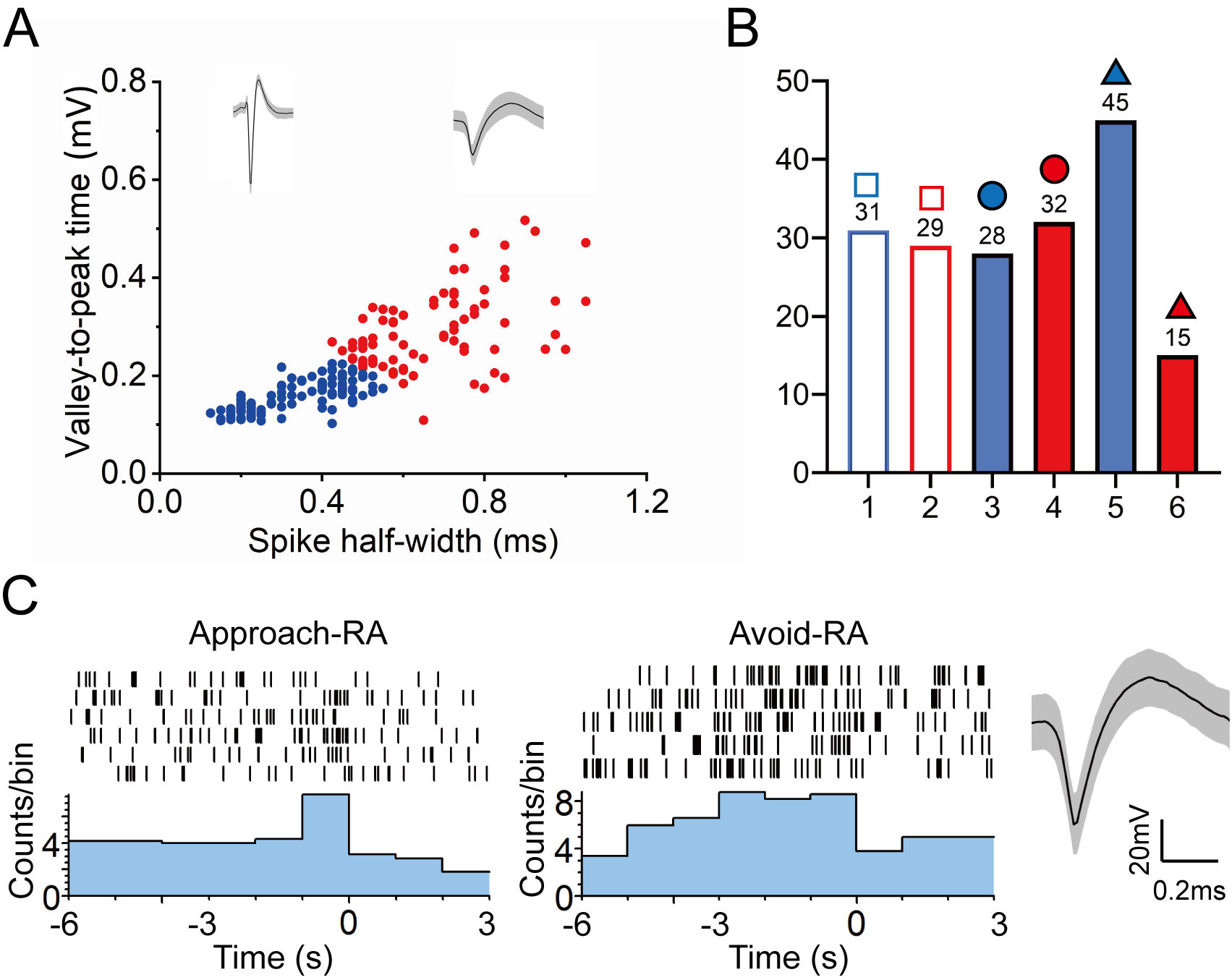

Supplement: Supplementary Figure 1 — Neuronal classification and activity responses. (A) HPC neurons (n = 180 neurons from 5 mice) were classified by valley-to-peak time and spike half-width when the mice entered the food zone; blue, putative GABAergic neurons; red, putative glutamatergic neurons; (B) Quantified neuronal responses to approach-RA, where blue hollow rectangle = no response GABAergic neurons, red hollow rectangle = no response glutamatergic neurons, blue triangle = approach-RA excited GABAergic neurons, red triangle = approach-RA excited glutamatergic neurons, blue circle = approach-RA inhibited GABAergic neurons, and red circle = approach-RA inhibited glutamatergic neurons; and (C) Representative raster plots and waveforms of dDG/CA3 neurons responding negatively to RA, in which RA-inhibited neurons exhibited a phasic increase in the firing rate before RA (in close arm, duration, ∼2–3 s) and a subsequent decrease of the firing rate during RA (in center zone). [file Image_1.tif]

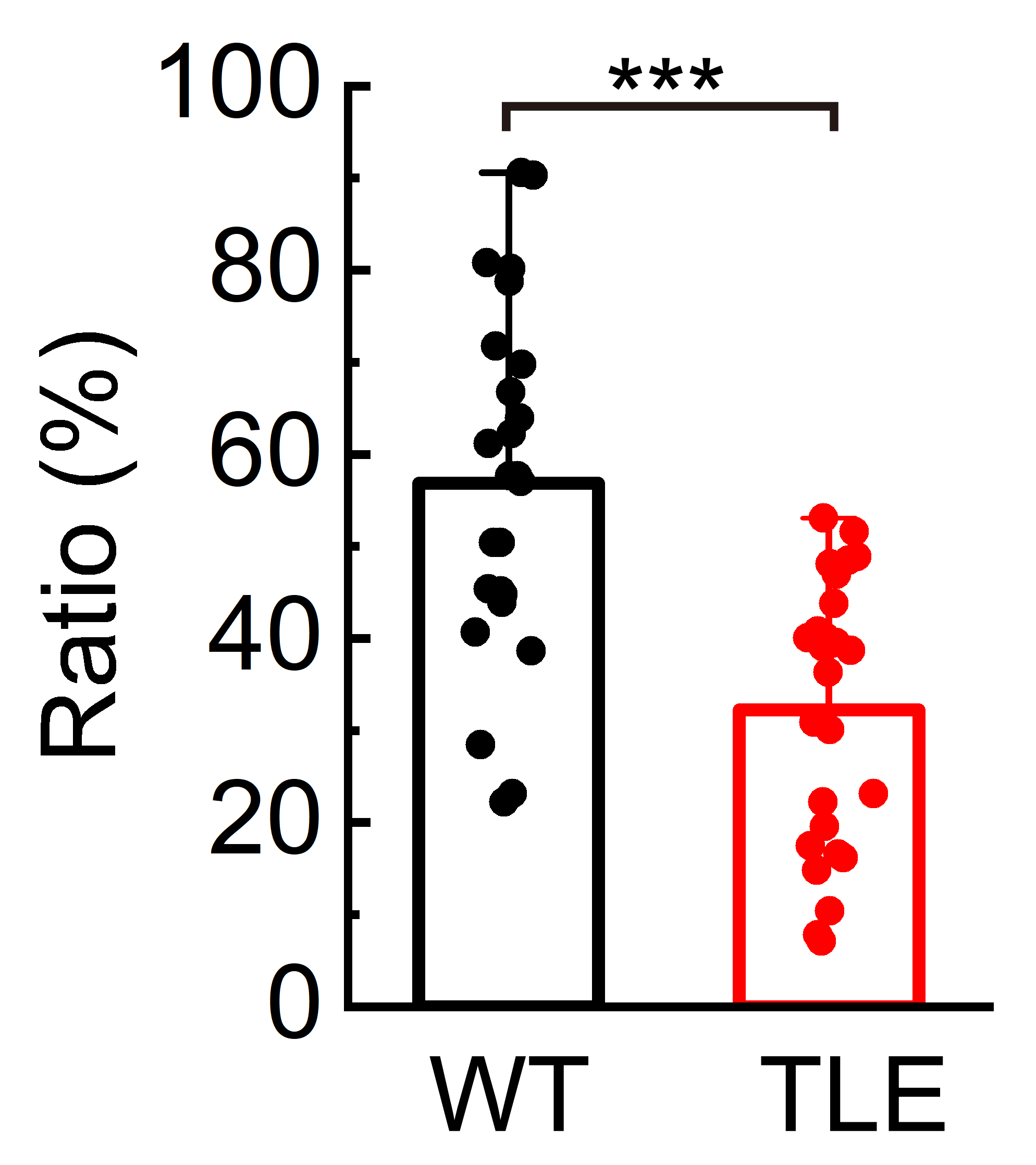

Supplement: Supplementary Figure 2 — The DAPI positive cells were reduced. The ratios were the fluorescence positive area (DAPI+ signal) divided area of ROI (region of interest) in dDG/CA3. WT, n = 22 slices from 6 mice, TLE, n = 27 slices from 6 mice. Error bars represent s.d. ***p < 0.001. Two-sample t-test. [file Image_2.tif]

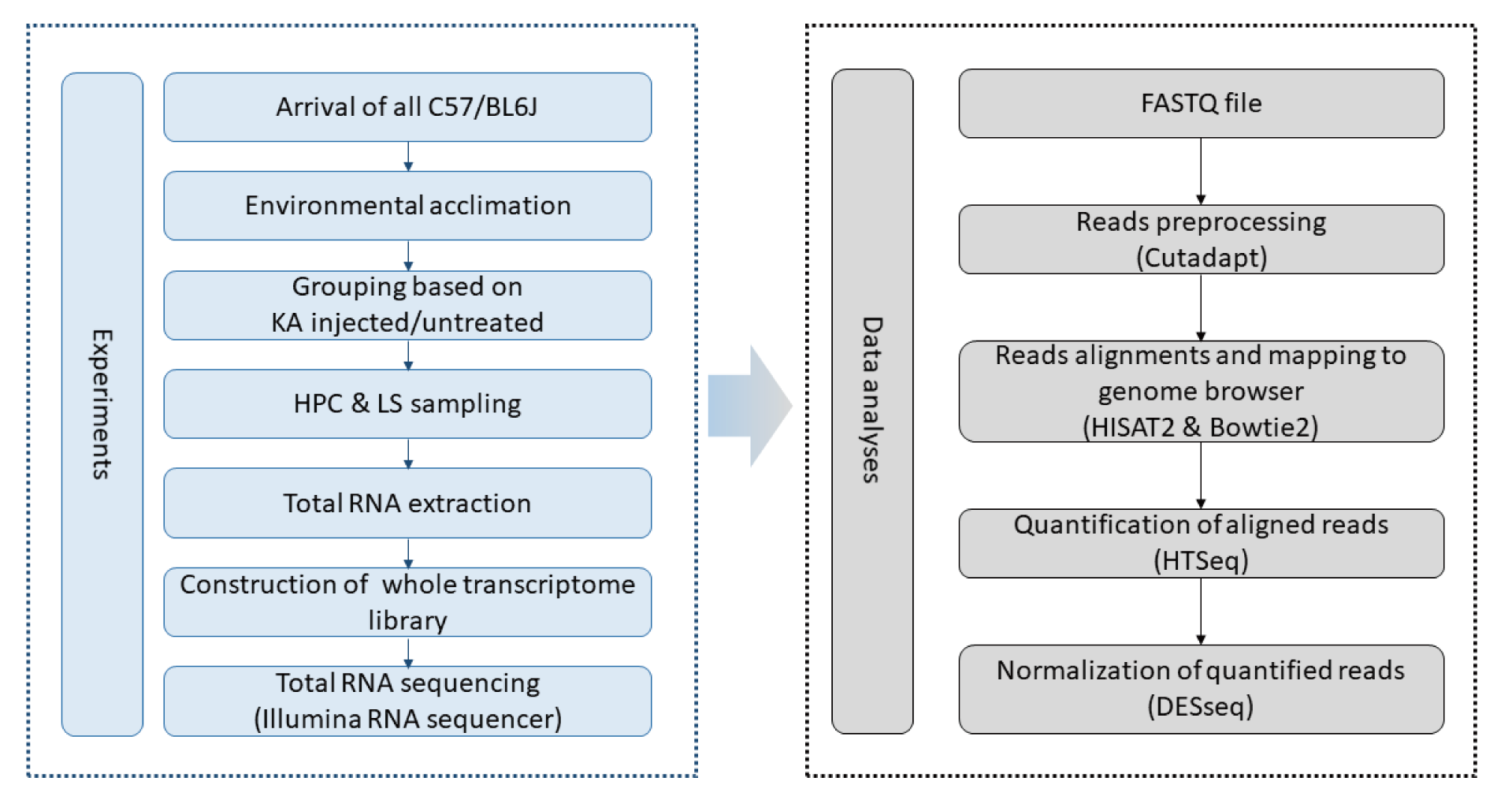

Supplement: Supplementary Figure 3 — Overview of RNA sequencing flow diagram. Wild-type 6-week-old C57BL/6J male mice were obtained and housed for 2-week environmental acclimation. Half of the wild-type 8-week-old mice were subjected to KA-injection. All mice were housed to 20-week-old for sampling. [file Image_3.tif]

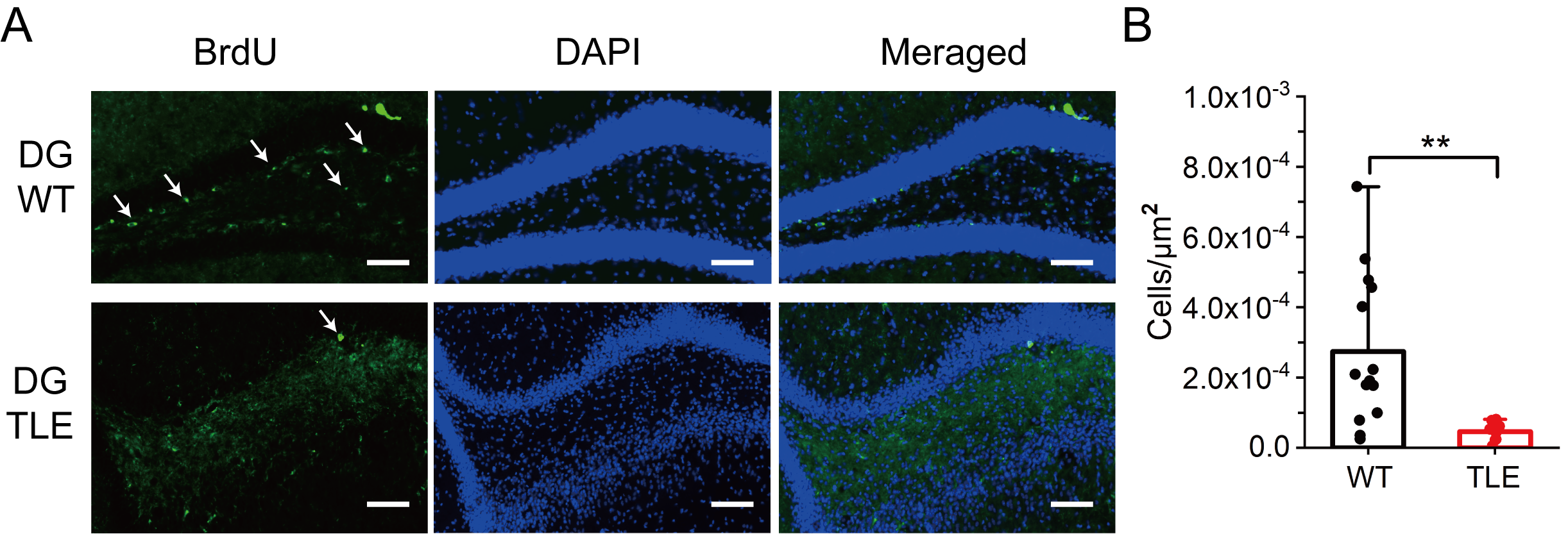

Supplement: Supplementary Figure 4 — Comparison of BrdU+ neurons in WT vs. TLE. (A) Representative images of immunostaining of BrdU+ neurons in dDG of WT and TLE mice. Scale bar, 50 μm. (B) The number of BrdU+ neurons in dDG (BrdU+: WT, n = 14 slices from 2 mice, TLE, n = 8 slices from 3 mice) (Error bars represent s.d; **p < 0.01, two-sample t test). [file Image_4.tif]

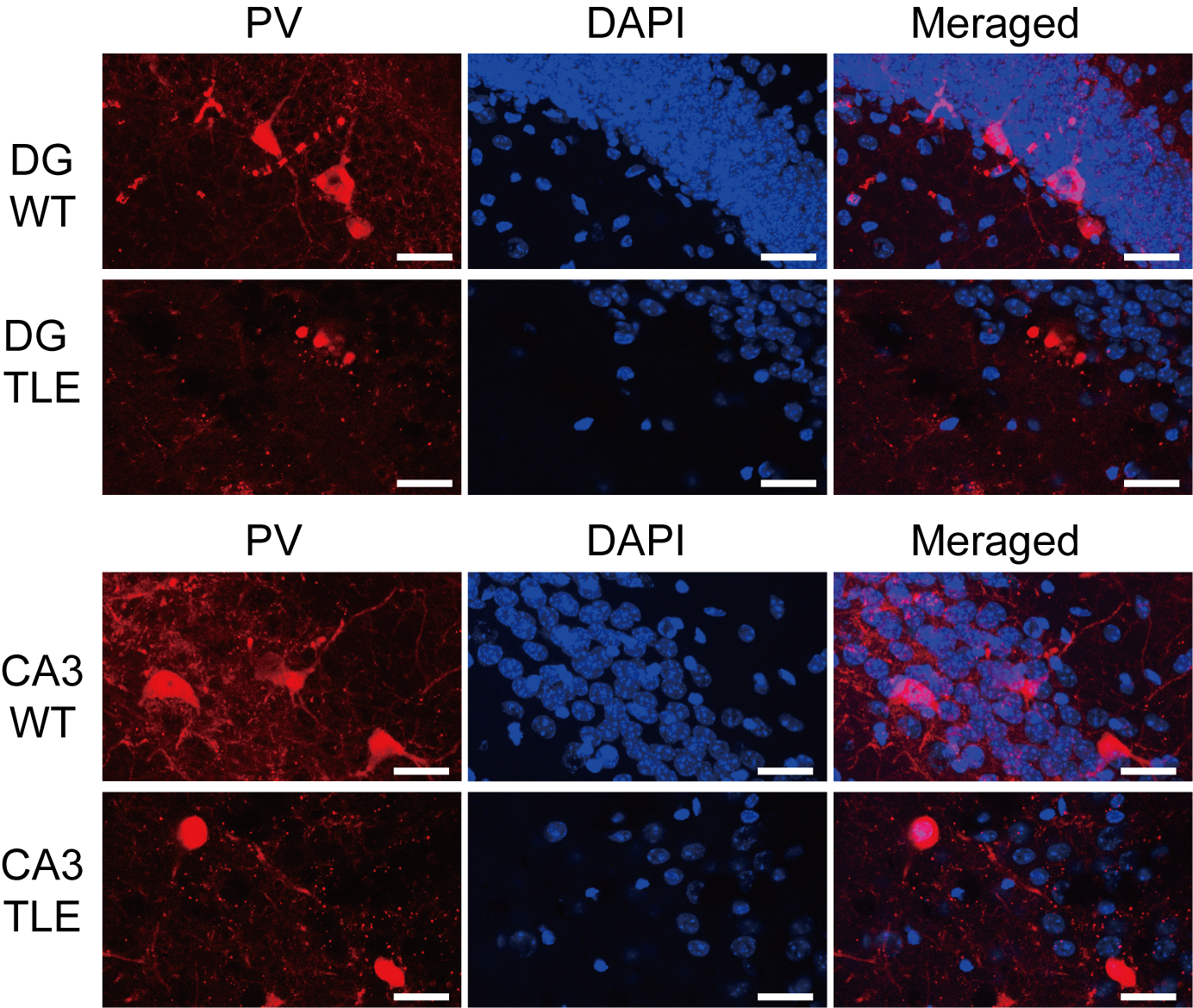

Supplement: Supplementary Figure 5 — Morphological changes in PV+ neurons. Representative confocal images at 40× showed that the morphology of PV+ neurons cell body altered in dDG/CA3 of TLE mice. Scale bar, 20 μm. [file Image_5.tif]

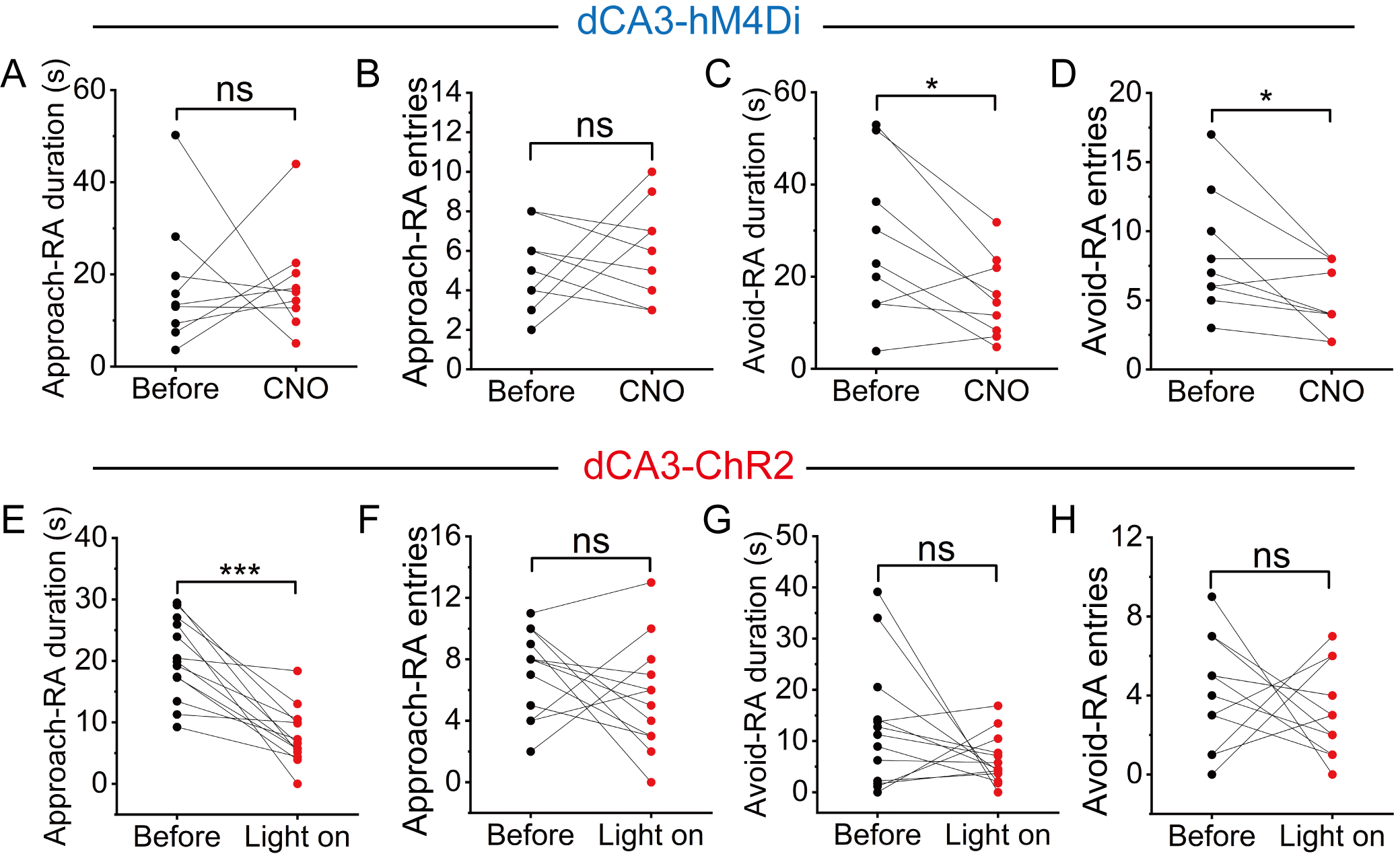

Supplement: Supplementary Figure 6 — Chemogenetics inhibition of hippocampal SOM+ neurons or optogenetic activation of hippocampal glutamatergic neurons impaired the RA. (A–D) Chemogenetics inhibition of hippocampal SOM+ neurons. (A) The approach-RA duration. (B) The approach-RA entries. (C) The avoid-RA duration. (D) The avoid-RA entries (before CNO, n = 9; CNO administration, n = 9). (E–H) Optogenetic activation of hippocampal glutamatergic neurons. (E) The approach-RA duration. (F) The approach-RA entries. (G) The avoid-RA duration. (H) The avoid-RA entries (before light, n = 12; light on, n = 12) (ns is no significant difference; error bars represent s.d; *p < 0.05; ***p < 0.001, paired-t test). [file Image_6.tif]
