# Supplementary material for: Dysfunction of the Hippocampal-Lateral Septal Circuit Impairs Risk Assessment in Epileptic Mice
Source: Front Mol Neurosci. 2022 Apr 29;15:828891. doi: 10.3389/fnmol.2022.828891 (PMC9103201; doi:10.3389/fnmol.2022.828891)
Supplement: Supplementary file 7 [file Table_1.docx]

**Supplementary Table 1 | Sample description.**

| **Sample #** | **Sample name** | **Sample description** | **Sex** | **Sample group** | **Brain region** |
| --- | --- | --- | --- | --- | --- |
| 1 | WT1 | 4-month-old WT | Male | WT | dHPC & LS |
| 2 | WT2 | 4-month-old WT | Male | WT | dHPC & LS |
| 3 | WT3 | 4-month-old WT | Male | WT | dHPC & LS |
| 4 | WT4 | 4-month-old WT | Male | WT | dHPC & LS |
| 5 | TLE1 | 4-month-old KA-induced TLE | Male | TLE | dHPC & LS |
| 6 | TLE2 | 4-month-old KA-induced TLE | Male | TLE | dHPC & LS |
| 7 | TLE3 | 4-month-old KA-induced TLE | Male | TLE | dHPC & LS |
| 8 | TLE4 | 4-month-old KA-induced TLE | Male | TLE | dHPC & LS |
| 9 | TLE5 | 4-month-old KA-induced TLE | Male | TLE | dHPC & LS |
| 10 | TLE6 | 4-month-old KA-induced TLE | Male | TLE | dHPC & LS |
